# Cancer Houses

**Published:** 1899-04-22

**Authors:** 


### CANCER HOUSES.

As to cancer houses, Mr. D'Arcy Power gives many striking instances of the repeated occurrence of the disease in the same building. He says: "We have thus localised epidemics of cancer to districts, to villages, and, by the following most interesting sequence, even to a single room. Miss B., aged 45, lived in a certain house in a suburb of London for 13 years, and died of cancer of the stomach in 1884. Miss T., aged 47, then succeeded to her place and occupied her bedroom. She had lived in the house for twenty years, and died of cancer of the liver in October, 1885. Mrs. J., aged 67, who had lived in the house for eight years, succeeded to the place and to the bedroom successively occupied by Miss B. and Miss T. Mrs. J. died of cancer of the breast and uterus in 1893. Each of these patients, my informant adds, appeared to be in perfect health until they took one another's place as 'housekeeper' to the barmaids of the establishment in which they had lived for so long a time. There was no blood relationship between them." Mr. D'Arcy Power thinks that the cases which he relates raise a strong presumption in favour of cancer being an infective disease to which some are more liable than others, and that locality seems to be a predominant factor in determining its incidence. But he does not think that the cause will be found in any given room, or house, or water supply, notwithstanding the curious cases which are related. It must, he thinks, be looked for much further afield, and it will almost certainly turn out that, much as is the case with malaria, there is some intermediate host which is necessary for the completion of the life cycle of the infective agent, and that it is the presence or absence of this intermediate host in the different districts under consideration which determines whether they shall or shall not become "cancer fields."

